# Manual of Veterinary Micro-Biology

**Published:** 1895-02

**Authors:** 


*Manual of Veterinary Micro-Biology.* By Professors MOSSELMAN and LIÉNAUX, National Veterinary College, Curghene, Belgium. Translated and edited by R. R. Dinwiddie, Fayetteville, Ark. Cincinnati: The Robert Clark Company. 1894.

The translation of this work by Dr. Dinwiddie will fill a long-felt want in the library of the busy practitioner of veterinary medicine. It is practical and instructive and contains nothing but what every veterinarian should desire to know. Too many practitioners are ignorant of the first rudiments of bacteriology, much to their disadvantage in this age of medicine. Bacteriology is bound to rule the medical world, and the earlier the general practitioner makes up his mind to this the quicker he will post himself upon the subject and the less likely will he be of becoming a back number. We take pleasure in recommending Dr. Dinwiddie's book, and wish for it a large circulation.

